# Incidence and Risk Factors of Ventilator-Associated Pneumonia among Patients with Delirium in the Intensive Care Unit: A Prospective Observational Study

**DOI:** 10.1155/2022/4826933

**Published:** 2022-01-13

**Authors:** Morteza Shamsizadeh, Ali Fathi Jouzdani, Farshid Rahimi-Bashar

**Affiliations:** ^1^Chronic Diseases (Home Care) Research Center, Hamadan University of Medical Sciences, Hamadan, Iran; ^2^Student Research Committee, Hamadan University of Medical Sciences, Hamadan, Iran; ^3^Anesthesia and Critical Care Department, Hamadan University of Medical Sciences, Hamadan, Iran

## Abstract

**Introduction:**

The incidence and risk factors for ventilator-related pneumonia (VAP) in patients with delirium are deficient, and there is a lack of in-depth knowledge of the impact of VAP on outcomes in this population. We investigated the incidence, risk factors, and outcomes of VAP in patients with delirium.

**Materials and Methods:**

This prospective observational study was performed in a surgical ICU at Be'sat Hospital in Hamadan, Iran, between 2018 and 2019. A total of 108 patients with delirium were identified using the Confusion Assessment Method (CAM) for the ICU and Intensive Care Delirium Screening Checklist (ICDSC) and enrolled in this study. The association between VAP and delirium, risk factors, and outcomes (ICU length of stay and ICU mortality) for VAP were investigated using the Cox proportional hazards model and logistic and simple linear regression analyses with a 95% confidence interval.

**Results:**

Of 108 delirium patients, 86 patients (79.6%) underwent mechanical ventilation (MV) and 16 patients (18.6%) experienced VAP during ICU stay. The median onset of VAP was 6.5 (IQR 4.2–7.7) days after intubation. Delirium patients with VAP stayed longer in the ICU (21.68 ± 4.26 vs.12.93 ± 1.71, *P* < 0.001) and also had higher ICU mortality (31.25% vs. 0%, *P* < 0.001) than subjects without VAP. According to multivariate cox regression, the expected HR for VAP was 53.5% lower for patients with early-onset delirium than in patients with late-onset delirium (HR: 0.465, 95% CI: 0.241–0.894, *P*=0.022). However, the expected hazard for VAP was 1.854 times and 4.604 times higher in patients with longer ICU stay (HR: 1.854, 95% CI: 1.689–3.059, *P*=0.032) and in patients with a prolonged MV duration (HR: 4.604, 95%CI: 1.567–6.708, *P*=0.023).

**Conclusion:**

According to the results, there seems to be an inverse relationship between early onset of delirium and VAP. This finding cannot be conclusively cited, and more studies in this filed should be conducted with a larger sample size. Furthermore, VAP in delirium patients is associated with increases in poor outcomes (higher ICU mortality) and the use of medical resources (longer stay in the ICU and MV duration).

## 1. Introduction

Delirium in the intensive care unit (ICU) is a common complication for critically ill patients due to the severity of illnesses, multiple comorbidities, multiorgan failure, drug interactions, old age, sleep deprivation, sedation, analgesia, and exposure to infections [1–3]. It is characterized by acute confusion, inattention, and either disorganized thinking or altered consciousness [4]. Delirium is associated with poor outcomes in hospitalized patients, including prolonged mechanical ventilation (MV), longer hospital and ICU length of stay (LOS), development of post-ICU cognitive impairment, higher mortality rate, and higher cost of care [5–7].

Previous studies reported an incidence of delirium in the ICU between 11% and 40% [8–10]. However, much higher incidence has been reported in intubated patients (reaching 80%) [11, 12]. Due to acute multisystem illnesses, comorbidities, medications, and many other risk factors, mechanically ventilated ICU patients are at high risk for the development of delirium [13, 14]. In this population, cognitive impairment has a negative impact on the main outcome indicators such as weaning from the ventilator, the development of ventilator-associated pneumonia (VAP), and ICU LOS [15, 16]. VAP is a type of nosocomial pneumonia developing 48 h or more after receiving MV [17]. VAP is the most common hospital-associated infection (HAI) among adult patients in the ICUs, with an incidence between 15% and 45% [18].

The incidence and risk factors for VAP in patients with delirium are deficient. In addition, there is a lack of in-depth knowledge of the impact of VAP on outcomes in this population. Thus, it is important to study and evaluate the factors and outcome associated with VAP in critically ill patients with delirium. Not only does this help us better understand critical care and closely monitor infections but also it helps us make decisions about the treatment and care of delirium patients at high risk of pneumonia. Hence, we conducted this prospective observational study to investigate the incidence, risk factors, and outcomes of VAP in patients with delirium, as well as the association between the onset of delirium and the onset of VAP.

## 2. Materials and Methods

### 2.1. Study Design and Participants

This was a prospective observational study performed in a surgical ICU at Be'sat Hospital in Hamadan, Iran, between 2018 and 2019. The study protocol was approved by the Ethics Committee of Hamadan University of Medical Sciences, Hamadan, Iran, with code IR.UMSHA.REC.1400.433. Due to the observational nature of this study, informed consent was exempted. This observational study was conducted and reported in accordance with the recommendations of the Strengthening the Reporting of Observational Studies in Epidemiology (STROBE) statement [19]. Patients with delirium were enrolled in the study, which was identified using the Confusion Assessment Method (CAM) for the ICU and Intensive Care Delirium Screening Checklist (ICDSC), who were admitted to the surgical ICU due to trauma (head, chest, and abdominal trauma). Delirious patients younger than 18 years of age and those who had developed pneumonia prior to initiation of MV were excluded from the study.

### 2.2. Delirium Assessment

To find patients with delirium, patients admitted to the surgical ICU due to trauma were assessed during each shift (three times daily; morning, noon, and evening) by trained nurses, using the Confusion Assessment Method for the ICU (CAM-ICU) [20] and Intensive Care Delirium Screening Checklist (ICDSC) screening tools [21]. If the patient exhibits acute changes or fluctuations (determined by abnormalities), more concentration and confusion, or changes in the level of consciousness during the course of their mental state, the CAM-ICU assessment is positive. In terms of ICDSC, the list is divided into eight categories, including the level of consciousness, inattention, disorientation, delusions, psychomotor agitation, inappropriate speech or mood, sleep disorders, and symptom fluctuations [22]. If the patient meets the listed criteria, each category is coded as present (a score of 1), with a maximum score of 8. At any time during the ICU hospitalization, patients with ICDSC score ≥4 were classified as having delusions. Those with all ICDSC scores <4 on all ratings were classified as having never had delirium.

### 2.3. Data Collection

A well-trained intensive-care physician is assigned for data collection (F.R-B). The demographic data collected include age and sex based on the medical records of all eligible ICU patients with delirium. The types of trauma (head, chest, and abdominal trauma), as the reasons for admission to the ICU, were gathered. Serum glucose levels for each patient were checked and recorded at the first day of ICU admission by analyzing a small amount of blood from a fingertip. The APACHE IV score was calculated in the first 24 hours of admission to the ICU [23, 24]. The level of sedation was assessed using the Richmond Agitation Sedation Scale (RASS) [25]. The RASS is a 10-point scale ranging from unarousable (–5 points) through calm (0 points) to combative (4 points) [26]. In addition, ICU length of stay (LOS) and the status (alive or death) of patients were recorded.

### 2.4. Outcomes

The primary outcome was VAP, and the diagnosis of VAP was identified using the Clinical Pulmonary Infection Score (CPIS) [27]. Patients who underwent MV were monitored and examined by an Infectious Disease specialist. According to chest X-ray, body temperature, white blood cell count, airway secretions, the ratio of arterial blood oxygen to inhaled oxygen, and respiratory culture and smear bacterial pneumonia index were calculated, and the patient was considered to have pneumonia if scored more than 6 [28]. The second outcome included length of stay in the ICU and ICU mortality. The data were collected before hospital discharge. If patient died during hospitalization, the data were collected at the day of death.

### 2.5. Statistical Analysis

Continuous variables including age, APACHE IV score, RASS score, serum glucose levels, onset of delirium, days of MV duration, onset of VAP, and ICU LOS are presented as means with standard deviations (SD) or medians and interquartile ranges (IQR 25–75%) when appropriate. The normality of data distribution was analyzed using the Shapiro–Wilk test. Differences between groups (with and without VAP) were analyzed using Student's *t*-test or Mann–Whitney U-test as appropriate. Categorical variables including gender, trauma types, and ICU mortality are reported as frequencies and percentages. Differences between groups were analyzed using the chi-square test or Fisher's exact test. Univariate and multivariate logistic regression was employed to estimate the odds ratio (OR) with 95% confidence interval (CI) to investigate the independence of risk factors for VAP in patients with delirium. To avoid overfitting in the multivariate model, just the factors which lead to *P* value less than 0.05 in univariate analysis were selected for the multivariate model. In addition, univariate and multivariate proportional hazard Cox regression, with VAP as the event and the time to onset of VAP, were applied to analyse the relationship between the time to VAP and risk factors. In the multivariate analyses, the significant variable selection modeling was reported as hazard ratio (HR) with 95% confidence interval (CI). The correlation between the onset of delirium and VAP was assessed using a simple linear regression and Pearson correlation coefficient test. All data were analyzed using the Statistical Package for the Social Sciences (SPSS) 21.0 statistical package (Chicago, IL, USA), and two-side *P* < 0.05 indicated a statistically significant difference.

## 3. Results

We enrolled 108 patients with delirium in the study with a median onset of delirium 4 (IQR 3-4) days after admission. Of the 108 delirium patients, 86 patients (79.6%) underwent MV and 16 patients (18.6%) experienced VAP during their ICU stay. The median MV duration was 12 (IQR 9–13) days. The median onset of VAP was 6.5 (IQR 4.2–7.7) days after intubation. Baseline demographic and clinical characteristics and outcomes between patients with and without VAP are presented in Table 1. There were no differences between patients with and without VAP in terms of gender, cause of ICU admission, RASS score, and serum glucose levels. However, delirious patients with VAP were significantly older than delirious subjects without VAP, and they also had higher APACHE IV score, longer ICU stay, and higher ICU mortality. Furthermore, patients who developed delirium at a later time in their ICU stay were more likely to develop VAP than those with early onset delirium (mean of onset of delirium 5.62 ± 1.70 vs. 4.17 ± 1.11, *P*=0.004.

According to the multivariate logistic regression analysis in Table 2, VAP was independently associated with age (odds ratio (OR) = 1.091, 95% confidence interval (CI): 1.022–1.985, *P*=0.041), onset of delirium (OR = 0.085, 95% CI: 0.017–0.432, *P*=0.003), MV duration (OR = 10.78, 95% CI: 1.876–15.544, *P*=0.023), and ICU LOS (OR = 3.904, 95% CI: 2.464–6.183, *P* < 0.001).

A proportional hazard Cox regression analysis with time-varying covariates, taking delirium as the event and the time to onset of ICU delirium, was used in the study, which are listed in Table 3. Based on multivariate cox regression, the expected hazard ratio (HR) for VAP is 53.5% lower for patients with early-onset delirium than in patients with late-onset delirium (HR: 0.465, 95% CI: 0.241–0.894, *P*=0.022). However, the expected hazard for VAP was 1.854 times and 4.604 times higher in patients with longer ICU stay (HR: 1.854, 95% CI: 1.689–3.059, *P*=0.032) and in patients with prolonged MV duration (HR: 4.604, 95% CI: 1.567–6.708, *P*=0.023).

A simple linear regression was carried out to test if the timing of the onset of delirium predicted development of VAP. The results of the regression indicated that the timing of the onset of delirium can predict significant (18.1%) development of VAP, *F*(1,106) = 23.35, *P* < 0.001. This regression model that has a significant variable but a low *R*^2^ (0.181) indicates that the onset of delirium as an independent variable is correlated with the onset of VAP as a dependent variable, but it does not explain much of the variability in the dependent variable (*β*_1_ = 0.73, *P* < 0.001). In addition, based on Pearson correlation coefficient test finding, the onset of VAP and the onset of delirium were moderately correlated with *r* = 0.425, *P* < 0.001.

## 4. Discussion

To our best knowledge, this study was the first study exploring the incidence, risk factors, and outcomes of VAP in patients with delirium. The overall incidence of VAP in patients with delirium was 18.6%. According to our results, age, onset of delirium, MV duration, and ICU length of stay were independent risk factors for VAP. Older age, prolonged ICU stay, and longer MV duration could increase the risk of VAP. Data from other studies also identified age [29] and longer ICU stay [30] to be risk factors for the occurrence of VAP, which is consistent with our findings.

**Figure 1:**
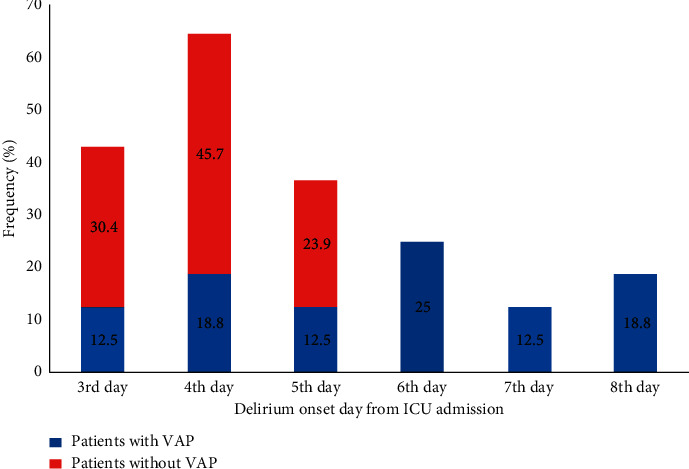
Comparison of early- versus late-onset delirium in patients with and without VAP (*P* < 0.05).

The most notable finding of this study is the early onset of delirium from ICU admission as a protective factor for VAP. The result indicated that the expected HR for VAP was 53.5% lower for patients with early-onset delirium than in patients with late-onset delirium. As shown in Figure 1, the highest incidence of delirium occurred on the fourth day after ICU admission (64.5%) followed by the third (49.2%) and fifth day (36.4%). But, the higher frequency in these three days is related to the patients without VAP. Interestingly, in more than half of the patients with VAP (56.3%), delirium occurred from the sixth day onwards. This could be due to the fact that the early onset of delirium in patients leads to critical care and receive more attention from the intensivist, which can have a protective effect on VAP. On the other hand, late onset of delirium when the patient needs MV or after that, due to the nature of delirium, which is associated with a greater period of MV and an increase in the ICU length of stay, can increase the risk of VAP [6, 31, 32]. According to our findings, the onset of delirium could predict only 18% of VAP incidence; this finding cannot be conclusively cited, and more studies in this field should be conducted with a larger sample size.

Critically ill ICU patients are subject to numerous risk factors for delirium, but the two risk factors that are almost generally experienced by ICU patients are exposure to sedative and analgesic medications and sleep deprivation [33]. Evidence has shown that, in critically ill patients with delirium, cognitive impairment can have a negative effect on weaning ventilator, VAP development, and length of stay in the ICU. On the other hand, patients with delirium are unable to cooperate with critical-care nurses to improve the quality of healthcare as well as infection prevention approaches that can reduce the incidence of VAP. Therefore, these patients are more prone to VAP and consequently have a higher mortality rate, which requires more attention.

Our study had several limitations. First, it was a single-center observational study performed in an ICU. Because we only enrolled trauma patients with delirium, the incidence of VAP may have been skewed. Second, as we only enrolled patients with delirium, it was not possible to compare the incidence of VAP in the two groups with delirium and without delirium. Third, we recorded only the onset of delirium and the onset of VAP and missed patient intubation time. Recording the time of intubation could help us to determine if delirium occurred before or after. Fourth, it was impossible to obtain daily sequential organ failure assessment (SOFA) scores because some of the variables used to calculate SOFA scores were unavailable.

## 5. Conclusions

In the present study, we investigated the incidence, risk factors, and outcomes of VAP in patients with delirium, demonstrating that patients with delirium have an 18.6% risk of developing VAP. We also found that VAP in delirious patients was associated with longer LOS in the ICU, prolonged MV duration, and higher ICU mortality rate. Based on the results found in the current study, in planning VAP prevention strategies, attention should be focused on patients with late-onset delirium and may have been close to or occurred after the intubation time. Further high-quality research with larger sample size is needed to strengthen the evidence base.

## Figures and Tables

**Table 1:** Baseline demographic and clinical characteristics and outcomes of patients with delirium according to with and without VAP (*n* = 108).

Variables		Patients without VAP (*n* = 92)	Patients with VAP (*n* = 16)	Total patients (*n* = 108)	*P* value
Age	Mean ± SD (years)	37.82 ± 10.96	51.06 ± 11.48	39.78 ± 11.96	<0.001^*∗*^
	Range (years)	(25–52)	(38–68)	(25–68)	

Gender	Male (%)	70 (76.1)	9 (56.3)	79 (73.1)	0.098
	Female (%)	22 (23.9)	7 (43.8)	29 (26.9)	

Cause of ICU admission	Head trauma (%)	78 (84.8)	15 (93.8)	93 (86.1)	0.462
	Chest trauma (%)	8 (8.7)	1 (6.3)	9 (8.3)	
	Abdominal trauma (%)	6 (6.5)	0	6 (5.6)	

Illness scoring systems	APACHE IV, mean ± SD	13.80 ± 1.65	19.87 ± 2.63	14.70 ± 2.82	<0.001^*∗*^
	RASS, mean ± SD	2.97 ± 0.79	2.93 ± 0.85	2.97 ± 0.81	0.852

Onset of delirium	Mean ± SD (days)	4.17 ± 1.11	5.62 ± 1.70	4.38 ± 1.31	0.004^*∗*^
Serum glucose levels	Mean ± SD (mg/dL)	174.89 ± 12.25	178.81 ± 27.36	175.47 ± 15.31	0.347
Outcomes	ICU LOS, mean ± SD (days)	12.93 ± 1.71	21.68 ± 4.26	14.23 ± 3.84	<0.001^*∗*^
	Mortality rate (%)	0	5 (31.25)	5 (4.6)	<0.001^*∗*^

VAP: ventilator-associated pneumonia; APACHE IV: Acute Physiology and Chronic Health Evaluation IV; RASS: Richmond Agitation-Sedation Scale; LOS: length of stay; ^*∗*^statistically significant <0.05.

**Table 2:** Univariate and multivariate logistic regression analysis to determine the independent factors associated with VAP in patients with delirium.

Variables	Univariate		Multivariate	
	OR (95% CI)	*P* value	OR (95% CI)	*P* value
Age	1.116 (1.050–1.185)	0.003^*∗*^	1.091 (1.022–1.985)	0.041^*∗*^
Gender (male vs. female)	2.475 (0.826–7.418)	0.106		
Trauma type (head vs. chest and abdominal)	0.371 (0.045–3.041)	0.356		
APACHE IV score	1.269 (1.191–1.352)	<0.001^*∗*^	1.122 (0.964–1.305)	0.138
RASS score	1.043 (0.568–1.915)	0.891		
Onset of delirium	0.109 (0.029–0.408)	<0.001^*∗*^	0.085 (0.017–0.432)	0.003^*∗*^
MV duration (days)	13.98 (10.82–27.25)	0.011^*∗*^	10.78 (1.876–15.544)	0.023^*∗*^
Serum glucose level	0.975 (0.944–1.007)	0.129		
ICU LOS	3.486 (2.505–4.852)	<0.001^*∗*^	3.904 (2.464–6.183)	<0.001^*∗*^

OR: odds ratio, ^*∗*^statistically significant <0.05; APACHE IV: Acute Physiology and Chronic Health Evaluation IV; RASS: Richmond Agitation-Sedation Scale; LOS: length of stay.

**Table 3:** Proportional hazard Cox regression analysis to determine the independent factors associated with VAP in patients with delirium.

Variables	Univariate		Multivariate	
	HR (95% CI)	*P* value	HR (95% CI)	*P* value
Age	1.021 (0.977–1.068)	0.353	0.987 (0.923–1.056)	0.713
Gender (male vs. female)	0.709 (0.247–2.031)	0.522	0.081 (0.009–1.745)	0.126
Trauma type (head vs. chest and abdominal)	0.211 (0.022–2.026)	0.177	0.381 (0.007–19.49)	0.631
APACHE IV score	1.072 (0.864–1.329)	0.530	1.283 (0.898–1.831)	0.171
RASS score	2.012 (0.916–3.986)	0.191	2.845 (0.965–8.387)	0.058
Onset of delirium	0.817 (0.303–0.906)	0.041^*∗*^	0.465 (0.241–0.894)	0.022^*∗*^
MV duration (days)	6.012 (1.916–10.906)	0.003^*∗*^	4.604 (1.567–6.708)	0.023^*∗*^
Serum glucose level	0.999 (0.976–1.022)	0.919	0.983 (0.945–1.023)	0.394
ICU LOS	2.894 (1.784–4.019)	<0.001^*∗*^	1.854 (1.689–3.059)	0.032^*∗*^

HR: hazard ratio, ^*∗*^statistically significant <0.05; APACHE IV: Acute Physiology and Chronic Health Evaluation IV; RASS: Richmond Agitation-Sedation Scale; LOS: length of stay.

## Data Availability

The data that support the findings of this study are available from the corresponding author upon reasonable request.
